# Hydrate of Chloral: A Therapeutic Study

**Published:** 1889-03

**Authors:** John Kent Spender

**Affiliations:** Physician to the Royal Mineral Water Hospital, Bath


THE BRISTOL

Medico-Chirurgical Journal.

MARCH, 1889.

### HYDRATE OF CHLORAL: A THERAPEUTIC STUDY.

BY

John Kent Spender, M.D. Lond.;

*Physician to the Royal Mineral Water Hospital, Bath.*

It was said by a great master, Samuel Taylor Coleridge, that every thinking and working man should take stock now and then of his intellectual material, test its force and action, and satisfy himself that it still has practical meaning and power. Those busy craftsmen, physicians and surgeons, ought to take earnest heed of Coleridge's saying. Things in use yesterday go out of fashion to-day, although they are as true and vital as ever; or the reasons for their original use fade from the memory; or a popular whim runs against them, and professional judgment bows unworthily to ignorant prejudice. The fluctuations of the therapeutic market make an interesting chapter of medical history. And we obey Coleridge's precept when we take a remedy of high value and importance, trace its chemical genealogy, estimate its relation to other remedies, and try to fix its just position in the cosmos of medical science.

For, the way to know how to use one medicine well, is to know how to use all other medicines well. To be a specialist in therapeutics, in the sense of knowing a great deal about the application of one medicine and very little about all others, is an empiricism not above the quack's level. The man of one book has been embalmed in a cynical proverb; but the man of one prescription is a creature not only ridiculous, but full of danger. Drawing an illustration from general literature, one secret of Professor Gardiner's success as the historian of the great Civil War lies in his freedom from insularity. He knows not only English affairs, but foreign affairs; and he knows the links which bind these two groups of affairs together. So with the therapeutist; he ought to know the links, physiological and pharmacological, which connect the anodynes with each other, and the whole group of anodynes with their therapeutic neighbours. For neighbours they are, and only a narrow "insularity" will make an idol of one drug and forget the honour due to every member of the therapeutic code.

Now, concerning the hydrate of chloral, the subject of our present study, we must allow that the promise of its magnificent dawn has not been altogether kept; and yet we hardly know why. If the healing properties attributed to chloral be true, they are true for ever; a law in therapeutics is as valid and lasting as a law in physiology. It is beyond the waves of human thought and the whims of human temper. Very unreasonable, therefore, are those oscillations of opinion, called fashion, which at one moment dignify a remedy with an ovation, and only a few years afterwards doom it to comparative neglect. Medical virtue once established, is a part of the things that are: prejudice and apathy cannot disestablish that virtue, or make as though it had never been discovered. Think what glory there was in hypodermic medication ten or twelve years ago, and how little in comparison it is used now! What unworthy stewardship this is! Such, however, are the devious paths of human thought and action, and so it ever will be.

Chloral* and chloro-acetic acid were discovered by Liebig in 1839. June 2, 1869, may be chronicled as the therapeutic birthday of chloral; and the thing was brought to light from the experience of Dr. Oscar Liebreich, then chemical assistant to the Pathological Institute of Berlin. In the *Charité* at Berlin the first experiment of chloral on man was made. It was administered subcutaneously to an insane person in very small doses. No local irritation followed; but the general effect was small. One dose of 20 grains by the mouth produced a sleep for five hours. In another patient, suffering from stupor and melancholia, 50 grains of chloral dissolved in water caused sleep for sixteen hours.

In July, 1869, Spencer Wells said that he had received two ounces of chloral, a substance which had been exhibited to the Medical Society of Berlin as a "new hypnotic and anæsthetic."

In the *Lancet* for October 2nd, 1869, the Editor writes that "chloral had become a subject of interest at a period when, in the absence of all other topics of a like kind, it could stand alone and invite universal attention. To this circumstance it owed an unusually fine introduction to

* Chemical abbreviation for "hydrate of chloral."

the medical world." Liebreich, it seems, imagined that chloral might supersede chloroform, bichloride of methylene, and ether. But by more judicious advocates the claim put forward for it was, not that it was an anæsthetic, but a rival to opium. One drachm of chloral was announced to be a "not immoderate dose" for an adult. It was observed that the "stupor or sleep" was attended with great muscular prostration and decrease of animal temperature; while sensibility was proportionately exalted. This "exaltation of sensibility" was specially insisted on by Demarquay and other French observers; but Spencer Wells's first experience of chloral was as an analgesic. In a "severe and intractable case of sciatica," defying huge quantities of hypodermic morphia and atropine, 30 grains of chloral "procured for the patient the best night she had enjoyed since the beginning of her illness—a night of perfectly tranquil sleep, from which she awoke fresh and well as from natural slumber."

At the Exeter meeting (1869) of the British Association for the Advancement of Science, Dr. B. Richardson presented a valuable experimental report. His postulate was, that the insensibility and the sleep which chloral produces are not intended to represent or to rival the action of the volatile anæsthetics which we use for the abolition of pain during surgical operations. Whatever of useful intention there might be in the introduction of chloral, was included in the virtue it possessed of causing prolonged sleep. The general result of its administration to vertebrate animals was to cause perfect sleep, without any preliminary excitement, sign of oppression, or disturbance. Dr. Richardson quotes, with apparent approval, the theory of the action of chloral as propounded by Liebreich. When it is treated with an alkali, it is resolved into chloroform and a formate. The blood is an alkaline fluid; therefore, says Liebreich, when chloral is introduced into the organism, every small particle of it will consume the surrounding quantity of alkali, and the decomposition will be completed only after the required amount of alkali has been furnished by the blood. In the smallest point of time the minimum quantity of chloroform is developed, and passes to the first place of action, the ganglia-cells of the cerebrum. With the increase of chloroform in the blood, the action extends to the ganglia of the spinal cord, and, lastly, to the ganglia-cells of the heart. The chloroform passes out of the body as chloroform, and is not resolved by oxidation into anything else. We may take it as established, therefore, that the agency at work is chloroform, chemically made within the body. But the really remarkable point is, that the nervous power of the heart is the last that suffers; and Richardson proved that in fatal cases the function of the heart is the last destroyed. It is well to recall a fact so contrary to modern teaching, which teaching is a perversion of the original doctrine of chloral.

Next came some surgical experience from the *clinique* of Langenbeck. By means of chloral he quieted delirium, following comminuted fracture of the humerus, in a woman of intemperate habits, after the utter failure of opium, morphia, and brandy. Langenbeck emphatically praises the "peaceful normal sleep" so quickly induced by chloral.

Sir James Simpson now appears upon the scene, with credentials which entitle him to be heard; and his confident oracle was, that the new remedy would prove of immense value in the practice of medicine, surgery, and midwifery. He confirms all the good things spoken of it, and he prescribed it for hysteralgia, dysmenorrhœa, and pleurodynia. As a pure hypnotic, Simpson gave chloral in the excessively large dose of 50 or 60 grains.

A few months later (March, 1870), chloral had advanced to the stage when it was called by high authority an "interesting drug." An "epidemic rage" for the new medicine had begun. It was time, therefore, for "the principal features of its action to be fairly made out." It came to be understood that the term *anæsthetic* could not be applied to chloral. The heavy and prolonged sleep produced by the largest dose is essentially different from anæsthesia. As a "producer of sleep," chloral was acknowledged to be inferior to opium alone; but the character of that sleep was superior to that caused by opium or any other drug. It was beginning to be recognised that not only unpleasant, but very dangerous, symptoms might result from such a dose as 50 grains. And, further, there was an indirect influence of chloral over pain. In chloral-sleep pain might be, so to speak, forgotten; but it could not be said that the pure and severe neuralgic type of pain was controlled.

It was incidentally remarked, about this time, that during the quiet slumber produced by chloral the inhalation of even a few minims of chloroform produces profound anæsthesia.

A paper by Dr. James Russell on the use of chloral in fever (*Glasgow Med. Journal*, February, 1870) was of much importance, and might be read now with interest and profit. When we give opium to a typhus patient, we combine two similar pathological tendencies; and we may precipitate into narcosis, functions previously going that way. Opium paralyses nerve- and muscle-tissues ("narcotic paralysis"). It is the almost perfect analogy between natural sleep and chloral-sleep which makes this drug so useful in febrile diseases. The condition of the pupil is the same in both cases. In all forms of sleep, the pupils expand the moment the sleeper is aroused and the eyes are opened. In opium-sleep, the pupil remains contracted. The chloral-sleeper may be roused at any time. He is at once in the full command of all his faculties and functions: he may take food, cough, expectorate, pass urine; and after these offices are fulfilled, he may fall again into unconsciousness. The patient may be said to be not properly awake; or, if he is, none of the excitement returns for which the chloral was given. The excretions are not affected by the administration of chloral. It far exceeds the bromide salts in efficiency and certainty as a hypnotic in fever, even when one of these salts is prescribed to the extent of a drachm every hour.

Dr. Russell's theory of the action of chloral was in harmony with Dr. Richardson's views. The alkalinity of the blood (as of other animal fluids) is increased by the typhus-fever process. The action of chloroform produced by the decomposition of chloral, represents the ultimate physiological action of chloral; and the chloral yields chloroform to the blood in exactly the way (so it is asserted) in which it might be expected to do the maximum of good and the minimum of harm. To the Edinburgh school of medicine, therefore, we are indebted for the scientific use of chloral in fever; and its beneficial application consists in preventing delirium by establishing normal sleep. The disease is not cut short, nor is its usual course affected in any material way.

In the autumn of 1870, cases were related by Crawford, Tuke, and Clouston to exemplify the usefulness of chloral in acute mania (puerperal or otherwise), puerperal convulsions, and as a remedy for the destructive habits in general paralysis of the insane.

About the same time, welcome news came from France in favour of the chloral treatment of traumatic tetanus. A case was given by M. Dufour, of Lausanne, in which a perfect recovery took place at the end of a month, an average of 12 grammes of chloral *per diem* having been administered. If it be contended that the patient got well by lapse of time, it must be allowed that the medicine exerted an invaluable remedial effect by reducing spasm, affording sleep, and permitting food to be taken. Departing for a moment from the strict chronology of our subject, we find (*Practitioner*, February, 1882) Mr. J. H. Salter treating a case of acute traumatic tetanus by an enormous dosage of potassium bromide and chloral, and with complete success. At one period the patient took half an ounce of the bromide salt and three drachms of chloral in twenty-four hours. In the course of twenty days, 60 drachms of chloral and 80 drachms of potassium bromide were consumed. For a time 5 to 10 grains of chloral were introduced hypodermically, in addition to the above, three or four times in the twenty-four hours; and the influence of these hypodermic doses remained a longer time than when the chloral was taken by the mouth.

Such was the store of evidence, gradually accumulating, which brought to chloral its high medical repute. The demonstration was slow but sure. Physicians looked upon their new gift as having secure and positive attributes, nearly all of which were on the good side of the account. Chloral seemed to be a pure and uncomplicated hypnotic. It was excellent for all disturbed and diseased conditions of the mind and nerve-centres attended with delirium (going on, perhaps, to mania), sleeplessness, and convulsions.* As an analgesic, its operation was uncertain; and when it acted as such, it might be in consequence of the sleep induced outlasting the pain, and rendering the patient insensible to it. In repeated small doses it was found to be a sedative for whooping-cough, spasmodic croup, and asthma. It was early observed, too, that in "head affections," with torpidity of bowels and retention of urine, chloral stimulates and helps the bowels and bladder to a more healthy action.

At the end of 1870, chloral was in the position of many illustrious people: being eulogised over-much, jealousies and alarms are raised. Every man and thing destined to become great must submit, in the first place, to the cruel ordeal of flattery; then to the more tonic discipline of criticism, and even reproach; and not until the man and the thing have been well weighed is it possible to form a just judgment about them. Was not chloral too good to be true? Must not such a splendid boon be counterbalanced by a thousand subtle banes? It is curious that Dr. B. Richardson, who was among the first prophets to arouse our enthusiasm, was among the first to quench it. In a lecture on Experimental and Practical Medicine (*Lancet*, February 11th, 1871), he notes that the period had already arrived when the medical use of chloral was lessening, and its popular use was increasing. The novelty of the drug, and the natural desire of testing for oneself, were the impulses that had moved medical men to prescribe chloral largely. "Chloral-drinking" had begun to prevail, with its train of serious

* In John Bunyan's allegory of the *Life and Death of Mr. Badman*, we read: "His doctor told him that his alarms had come from an affection of the brain, caused by want of sleep; they were nothing but vapours and the effects of his distemper." Now, Mr. Badman's "distemper" would have been speedily cured by chloral.

and irregular symptoms. Three classes of people were said specially to resort to chloral: alcoholic devotees; sufferers from neuralgia and other painful chronic diseases; and persons who have much grief or intellectual care. Nor was the late Dr. H. W. Fuller less emphatic in his warning, and he quite frightened the profession by the story of two cases. One person, in an advanced stage ("anasarca and bronchitis") of chronic Bright's disease, nearly died after taking the usual chloral-draught of the hospital, which contained 30 grains; and another patient (seen in consultation), described as a "young lady, aged 20, who was previously in fair health," died within eleven hours after taking the same quantity of chloral. Practitioners in America, too, reported that the long-continued use of chloral produced toxical effects somewhat like those produced by ergot.

Thus were doubt and uneasiness sown among the fraternity of medicine. But out of chaos Order is sure to come, and out of darkness Light, if men are earnest in the search for truth and human in their desires and sympathies. Here was a medicine which appeals to the nervous system when it is out of tune and time: let us group the phenomena of that system according to its physiological anatomy, and the therapeutic laws of chloral almost group themselves. The disorders of ceration include all deviations from mental health; motorial disorders include every disturbance of muscle, or interruption of the will in commanding muscle; and pain is the terrible emblem of perverted sensory forces. What can chloral do for all or any of these? What message does it bring for the healing of our patients?

It was an early and seductive tradition of chloral, that in "brain cases," however wild the delirium and however acute the mania, in a very short time there may be calm repose. But even in 1879 the value of chloral in treating *delirium tremens* was so little known or understood, that Dr. George Balfour then spoke of it as far from being established in medical opinion. (*Lancet*, February 1st, 1879.) He relates the first case of *delirium tremens* submitted to chloral-treatment in the Edinburgh Royal Infirmary. The man had been ill for a fortnight, and was ferociously mad. Two doses (30 grains each) of chloral, with an hour's interval between them, brought sound sleep, from which the maniac awoke a rational being. Similar success attended the chloral-treatment of the disorder in all other cases (sometimes 45 grains for a dose were administered). We can hardly imagine the old days when Sir Thomas Watson and Dr. Todd recommended,* in sheer despair, a "hair of the dog that bit him" (*i.e.* more alcohol) as a remedy for a morbid condition which alcohol had caused. I should grieve to misunderstand my veteran tutor and teacher, Dr. George Johnson; but in his admirable *Medical Lectures and Essays* his voice has an equivocal note in it when, writing of alcoholic delirium and praising the utility of chloral, he adds: "Opium is a valuable aid in the treatment, and will often cut short the disease." And again: "Some form of alcoholic stimulant may calm nervous excitement and procure sleep." The precepts of our living masters always deserve attention; but Dr. Johnson's views, now quoted, are at variance with actual therapeutic practice. Observe the problem: We are trying to get a patient out of the pseudo-narcosis of alcohol, and we are coaxing him to take food; ought we

* I heard Dr. Todd's "clinical lecture" at King's College Hospital in which this advice was given.

to give a medicine (such as opium) which will throw him back into narcosis, stop the cleansing of the blood by the excretions, and spoil the little appetite he has? And then to offer him more alcohol—is it not to add poison to poison?

In this delirium of tremors, the absence of sleep is both cause and consequence. Now comes the chloral with its gracious help: sleep is only a natural anæsthesia; and if it does not come by nature, it must be brought by art. Sleep is never a narcosis (in the language of pharmacology), though it may wear the likeness of narcosis: an appropriate formula for sleep would be, that the body is kept very calm and still for the purposes of nutrition, and of repairing wear and waste. The riot and fury of alcoholic delirium shake the tenement to pieces: it is not the mind only that raves, but the mind tears the body as well. Sleep is therefore all the more necessary to restore shattered muscles and nerve-centres; and chloral-sleep is the most perfect mimicry of nature-sleep which medicine can bring.*

In October, 1886, I was called to treat a lady severely ill with the horrors and terrors of alcoholic poisoning.† About her collateral symptoms, it is only needful to say that she had diligently soaked her tissues for years with brandy or wine; but from all this misdirected industry no organic degradation was apparent, except a syncopal tendency from heart-failure. The urine was free from albumen. Being even then a partial slave of the shallow fallacy that chloral might injure the heart more than

* Further, sleep restores the nerve-energy which is wanted for the digestion of food. Without a sufficiency of food, there is a danger of fatal collapse at any moment from stoppage of the heart's action.

† In the management of this case, I acknowledge with gratitude the assistance rendered by the Clifton Nursing Institution.

sleeplessness, I tried at first to cherish sleep by a large quantity of warm liquid food only. The frenzy was not in the least allayed. Between October 24th (the first day of medicinal treatment) and November 2nd, both inclusive, 13 drachms and 20 grains of chloral were administered in the following way: at first, 20 grains every three hours; then the same quantity every four or five hours; on October 28th, every eight hours; and after that at irregular intervals, according to urgency. At the earliest stage of treatment there came tranquillity; then profound, though broken, sleep; the sleep became more continuous with perseverance in the chloral: by degrees the medicine was withdrawn, and the waking intervals were more and more sane; food was plentifully taken in the shape of eggs, broths, and milk with soda-water *ad libitum*. Ordinary health seemed quite restored on the 4th of November. Another attack of the same kind of illness (in 1887) was treated successfully in the same way. Since then our supervision has been watching and preventive; and we find that when our patient is managed like the British soldier, and kept poor in purse, she abounds in health.

For the special delirium of acute alcoholic intoxication, chloral is the supreme remedy. My contention is, that there is no room for a scrap of doubt about it. A medical man who neglects it, or declines to use it, sins against certain knowledge; nor is he less a sinner if he goes back to opium or digitalis, or any other empirical stuff which has been luckily buried and forgotten. The absolute certainties of medical art are mournfully few, and we ought so much the more to value those we have. One would have expected that the splendid and accurate *Principles and Practice of Medicine* by the late Dr. Hilton Fagge would have made it clear, beyond all cavil, that the chloral-treatment of the so-called *delirium tremens* is the one anchor which we may nearly always trust; and our disappointment is great when we find it spoken of merely as having been recommended by the late Dr. Anstie fifteen or sixteen years ago. No particular praise is bestowed upon it. In due time our text-books of Materia Medica and Therapeutics will tell the student less about the dull chemistry and botany of his drugs, and more about their application to the exigencies of disease. It will not be thought sufficient, for instance, to say that "chloral is valuable" for this or that, without saying how and when it is to be prescribed, and what are the limitations of its use.

Motor disturbances go along with delirium so commonly, that the transference is easy when we pass to spasm and convulsion occurring without delirium. There is plenty of material here for the beneficent exercise of chloral. Let us consider, firstly, a form of convulsive disorder seen in children: the fits are very frequent (20 or 30 in the twenty-four hours), but short in duration, and singularly abrupt and sudden both in onset and termination. They are attended with absolute loss of consciousness. Now, it was pointed out by Dr. Rayne (*Lancet*, August 26th, 1876) that the muscular phenomena of these "fits" correspond very closely with those artificially obtained by Professor Ferrier on stimulating certain frontal and parietal convolutions of some of the lower animals. In a typical case narrated by Dr. Rayne, the patient, a boy aged five, enjoyed good general health, and the attacks were attributed to a blow upon the head. Chloral brought a speedy cure, after time had been lost in the prescription of what were called (with a touch of sarcasm) the "usual remedies."

I refer again to the therapeutics of tetanus, in order to quote the high authority of Dr. Lauder Brunton in favour of large doses of chloral. (*Practitioner*, August, 1877.) He says that they may be given without fear, and that the treatment is most valuable.*

In the neural terminology of Dr. Hilton Fagge, chorea is the ideal of the co-ordinated spasm.† In the acute grades of this nerve-storm, experienced therapeutists differ in their advice. The beginning of the chloral-treatment of chorea is associated with what was nearly a tragedy. To a girl, aged eight, suffering from the disease in an aggravated form, a nurse gave by mistake one night 45 grains of chloral: most alarming symptoms ensued, and the life of the little patient was in extreme jeopardy for some hours. There was an immediate improvement, however, in the choreic symptoms; and in the course of another day there was a complete and permanent cure. Such is the story published by Dr. Gairdner in the *Glasgow Medical Journal* for 1870. Emboldened by this happy accident, Dr. Bridges (*Practitioner*, March, 1877) urged that chloral should be given for chorea in doses that will cause sound sleep. The principle guiding us ought to be this: the patient must have ten hours' sleep in the twenty-four. The disease is naturally suspended during sleep, and therefore a drug which produces sleep is plainly indicated. A dose of ʒss. is administered; this may have to be repeated, or half as much, or less, if the patient does not sleep. Immediately on awaking another

* See also some cases under the care of M. Gueniot (*British Med. Journal*, October 6th, 1877).

† ". . . that distemper which impels the nerves
To motion without will; a dance 'tis call'd,
Of which Saint Vitus is the dancing-master."

WALTER SAVAGE LANDOR, *Last Fruit off an Old Tree*, p. 417.

dose is given, less or more as the patient had slept well or ill; and on waking again, another dose, less than the one before, and so until the amount of sleep required had been obtained. The chloral is then discontinued until the following night. By this ingenious plan Dr. Bridges tells us that he cured two bad cases—the first within twenty-four hours, the other within three days. Dr. Day reports rapid success in acute chorea by prescribing 10 grains of chloral every two hours.*

The far-reaching potency of chloral is shown in unexpected ways. A girl (daughter of a tradesman), aged now about 17, has been under my care for three or four years on account of epileptic seizures, more or less grave, and occurring at irregular intervals. Great benefit resulted from trustworthy medicines, notably a combination of sesquicarbonate of ammonium and bromide of ammonium; but there were fluctuations and some severe disappointments. The addition of 10 grains of chloral to the old nightly dose of the ammonium-salts was so successful, that the patient has had no epileptoid attacks since.

In an address on Analgesics delivered in 1886,† I ventured to suggest a way of administering chloral which might mitigate pain of a certain kind. Five grains dissolved in water, with a little syrup of tolu, should be given every hour for four or five doses, and then less frequently according to their effect. Since then the subject has had my constant attention. There is no pretension to rivalry between opium and chloral: a

* Dr. Althaus' view of chorea was, that it is an active hyperæmia of the corpora striata and neighbouring parts; and that chloral may cause anæmia of these structures.

† To the Therapeutic Section of the British Medical Association, at its Brighton Meeting.

sharp, decisive pain afflicting one nerve, or one small area of nerve-supply, lies within the jurisdiction of opium or of quinine, and nothing else. But neuralgia has many first-cousins. The "scalding" and "burning" of osteo-arthritis, when it affects the hands and wrists, are greatly relieved by chloral. From 15 to 20 grains at bed-time, with the liberty to take 8 or 10 grains more (if needed) at three or four o'clock in the morning, are to many sufferers a grateful boon, which is highly appreciated in our Mineral Water Hospital.

Chloral has been a great deal used for the migraine type of headache; but the literature of this therapeutic function is so large as to forbid the luxury of quotation.

So long ago as June, 1870, the late Mr. Weedon Cooke published records of cases in which 10 grains of chloral were given three times a day, to relieve the pain of malignant disease. And there can be no doubt of the utility of this method before that stage of misery is reached when our only resource is hypodermic morphine.

The administration of chloral in obstetric medicine has been illustrated by Dr. Playfair, and also by the late Sir James Simpson. The sponsor of chloroform would be naturally biassed in favour of chloral*; and he emphatically maintained that chloral lessens the pangs of labour, without restraining contractions of the uterus. Dr. Playfair says practically the same thing. He considers that chloral is applicable chiefly at a period of labour when we should not allow the inhalation of chloroform—namely, towards the end of the first stage. A mixture should be prepared, containing one drachm and a-half of chloral in six fluid ounces of water; of this,

* Simpson remarked incidentally that people suffering from irritable bladder and chronic cystitis derive much more benefit from chloral than from comparatively larger doses of opium.

one ounce is the standard dose, which may be repeated after twenty or thirty minutes. If the patient becomes drowsy, a third dose need not be given for an hour or an hour and a-half; and under no circumstances is more than one drachm of chloral to be taken during the course of a labour.

We will now briefly discuss the sedative* effect of chloral on two great physiological systems—the Respiratory and the Digestive.

The whole respiratory tract is submissive to the sedative power of chloral. Dr. George Johnson praises it for the spasm of *laryngismus stridulus*;† and for quieting the struggles of inflammatory croup, no combination excels chloral and ipecacuanha. The capacity of chloral to prevent the asthmatic paroxysm is acknowledged by all, and it may be combined with belladonna and iodide of sodium. In many shades of dyspnœa which have asthmatic affinities, especially when accompanied by a tinge of cyanosis on the skin, the hurtfulness of opium is a measure of the charm of chloral. In whooping-cough, chloral may indirectly check serious head and chest complications, by mitigating the convulsions and soothing nerve-irritability. Much larger doses must be given than have been usually recommended. For cough of every kind, especially that which attends bronchitis and pulmonary consumption, chloral should be tried; but of course it sometimes fails, and morphine may be the only thing useful. Chloral lessens the quantity of expectoration in all sorts of bronchial troubles. A short paper on "Chloral in Phthisis"

* The word *sedative* is used in the sense which the late Dr. Anstie made classical.

† Dr. Hughlings Jackson speaks of *laryngismus stridulus* as a "respiratory convulsion," and recommends chloral as the best remedy. His paper in *Brain* (Vol. II., p. 18) is well worth going through, but it is tough reading!

was one of the last contributions to practical medicine by the late Professor Hughes Bennett, and he spoke of its efficacy in arresting night-sweats.

It is possible that there has been some exaggeration about the power of chloral over diseases and disorders of the alimentary tract. But in my many notes, gathered from many sources, I find that for "agonising pain in the abdominal viscera," of uncertain origin, one drachm of chloral has been dissolved in fluid starch and administered as an enema. Given by the mouth, chloral has been said to allay the irritable diarrhœa of tubercular ulceration; and testimony has come from several quarters that it relieves the obstinate vomiting of organic disease and of pregnancy. We cannot doubt the anodyne value of chloral in the treatment of gastralgia, lead colic, and certain forms of dysmenorrhœa; but the agony of the passage of calculi (hepatic or renal) is not within its reach.

The evidence is tolerably unanimous that the therapeutic virtue of chloral does not suffer by frequent repetition.

There is an ethical element in chloral, alcohol, and opium which demands a weighty consideration. The perverted use of these substances may destroy self-control, and finally overthrow the moral and intellectual faculties. And so the casuists say that a huge responsibility rests on the adviser who prescribes chloral or alcohol or opium, lest haply they may be abused through ignorance or morbid craving. Undoubtedly. But the man who does not shrink from duty is never afraid of responsibility. If the necessity arises, the necessity must be met. The wise physician studies the "environments" of his client, and especially the force of his character.

The "poison of misused wine" has been the theme of poetry as well as the lament of psychology; and who would deliberately put the weak man or woman in the way of temptation? But if we are to strike out of the Pharmacopœia all the things which weak men and women have abused, our best traditions are made of none effect, and the reign of Timid Medicine begins.

It was said of a certain man that, although not a wit himself, he "rang the bell which called other wits together." My humble office in this paper has been to "ring a bell," and to assemble our therapeutic friends for a brief parley. Before this honourable convocation my plea would be: Here is a thing, rich and fine, wrested from the secret storehouse of Nature, and endowed with curious powers of healing. It has been challenged on all sides; exalted to the firmament, and then comparatively neglected. It has received the scrutiny of the keenest judgment, and passed the ordeal of the ripest experience. Within limits that every day become clearer, the medical application of chloral is now a postulate of the first order—a fact of the highest importance and interest. We are sure of our possession; we cannot and dare not pass it by. And it is "meet and right" so to employ it that it may, as far as possible, lighten and brighten the dark drama of human suffering.

